# Supplementary material for: The structural context of mutations in proteins predicts their effect on antibiotic resistance
Source: eLife. 2026 Oct 9;14:RP109450. doi: 10.7554/eLife.109450 (PMC13652538; doi:10.7554/eLife.109450)
Supplement: Peer review history

# Editor report: eLife Assessment

**DOI:** 10.7554/eLife.109450.3.sa0

**Authors:** Bryan D Bryson

**Affiliations:** Massachusetts Institute of Technology United States

**Keywords:** Compelling, Important

This **important** study leverages a large global dataset of tens of thousands of tuberculosis samples to place recurrent protein-coding mutations into their three-dimensional structural context, offering an expanded view of how antibiotic resistance emerges compared to traditional genetic analyses alone. The strength of evidence is **compelling**, supported by the scale and breadth of the dataset and the systematic structural analysis, although some of the assumptions made in the modeling approach are only partially supported. Overall, the work will be of broad interest to researchers studying microbial evolution, antibiotic resistance, and structure-function relationships in pathogens.

# Referee report: Reviewer #1 (Public review)

**DOI:** 10.7554/eLife.109450.3.sa1

Summary:

In this manuscript, Green et al. attempt to use large-scale protein structure analysis to find signals of selection and clustering related to antibiotic resistance. This was applied to the whole proteome of *Mycobacterium tuberculosis*, with a specific focus on the smaller set of known antibiotic-resistance-related proteins.

Strengths:

The use of geospatial analysis to detect signals of selection and clustering on the structural level is really intriguing. This could have a wider use beyond the AMR-focussed work here and could be applied to a more general evolutionary analysis context. Much of the strength of this work lies in breaking ground into this structural evolution space, something rarely seen in such pathogen data. Additional further research can be done to build on this foundation, and the work presented here will be important for the field.

The size of the dataset and use of protein structure prediction via AlphaFold, giving such a consistent signal within the dataset, is also of great interest and shows the power of these approaches to allow us to integrate protein structure more confidently into evolution and selection analyses.

Comments on revised version.

All my comments from the previous round of reviews have been addressed.

# Author comment: Author response

**DOI:** 10.7554/eLife.109450.3.sa2

**Authors:** Anna G Green, Mahbuba Tasmin, Roger Vargas, Maha Reda Farhat

**Affiliations:** University of Massachusetts Amherst Amherst United States; University of Massachusetts Amherst Amherst United States; Harvard Medical School Boston United States; Harvard Medical School Boston United States

The following is the authors’ response to the original reviews.

**eLife Assessment**

This valuable study leverages a large global dataset of tens of thousands of tuberculosis samples to place recurrent protein-coding mutations into their three-dimensional structural context, offering an expanded view of how antibiotic resistance emerges compared to traditional genetic analyses alone. The strength of evidence is convincing, supported by the scale and breadth of the dataset and the systematic structural analysis, although some of the assumptions made in the the modeling approach are only partially supported. Overall, the work will be of broad interest to researchers studying microbial evolution, antibiotic resistance, and structure-function relationships in pathogens.

We thank the reviewers and editors for their careful critique of our work. We believe the work has been strengthened by addressing the comments and are delighted to submit a revised version. This version has a detailed discussion of prior literature on structure analysis of antibiotic resistance variants in *Mycobacterium tuberculosis*, more details on dataset origins and data processing to improve reproducibility, better explanations of the evolutionary assumptions underlying the scoring method we developed, and more discussion of proteins that show homoplasic and clustered mutational signals that are not known to confer antibiotic resistance. We include detailed responses to the comments below.

**Public Reviews:**

**Reviewer #1 (Public review):**

Summary:

In this manuscript, Green et al. attempt to use large-scale protein structure analysis to find signals of selection and clustering related to antibiotic resistance. This was applied to the whole proteome of *Mycobacterium tuberculosis*, with a specific focus on the smaller set of known antibiotic-resistance-related proteins.

Strengths:

The use of geospatial analysis to detect signals of selection and clustering on the structural level is really intriguing. This could have a wider use beyond the AMR focussed work here and could be applied to a more general evolutionary analysis context. Much of the strength of this work lies in breaking ground into this structural evolution space, something rarely seen in such pathogen data. Additional further research can be done to build on this foundation, and the work presented here will be important for the field.

The size of the dataset and use of protein structure prediction via AlphaFold, giving such a consistent signal within the dataset, is also of great interest and shows the power of these approaches to allow us to integrate protein structure more confidently into evolution and selection analyses.

Weaknesses:

There are several issues with the evolutionary analysis and assumptions made in the paper, which perhaps overstate the findings, or require refining to take into account other factors that may be at play.

(1) The focus on antimicrobial resistance (AMR) throughout the paper contains the findings within that lens. This results in a few different weaknesses:

(a) While the large size of the analysis is highlighted in the abstract and elsewhere, in reality, only a few proteins are studied in depth. These are proteins already associated with AMR by many other studies, somewhat retreading old ground and reducing the novelty.

(b) Beyond the AMR-associated proteins, the proteome work is of great interest, but only casually interrogated and only in the context of AMR. There appears to be an assumption that all signals of positive selection detected are related to AMR, whereas something like cas10 is part of the CRISPR machinery, a set of proteins often under positive selection, and thus unlikely to be AMR-related.

We agree that environmental pressures beyond AMR may impose positive selection. In response to the reviewer’s comment we have now included more results and a supplementary figure of the findings about Cas10. We have expanded our results about the proteins found with significant clustering that are not known AMR-associated proteins, and clarified in the discussion that we don’t believe positive selection is caused only by AMR.

We note that data from homoplasic substitutions in proteins (Figure 1) does indeed support that AMR is the strongest driver of positive selection in the MTBC. A challenge is that knowledge about protein function varies in depth by protein, and in the MTBC the AMR proteins are among the most well-studied in the proteome. Thus, explanations from literature are most readily available for AMR proteins. Moreover, proteins may exhibit positive selection for more than one reason – for example, we find significant clustering in the proteins GlmM, GlmS, GlmU, and MurA, all of which are involved in amino sugar metabolism, a key component of the cell wall. These proteins could plausibly have a role in AMR via cell wall permeability mechanisms, and could plausibly have a role in adaptation to host environment via the same (or other) mechanisms.

(2) The strength of the signal from the structural information and the novelty of the structural incorporation into prediction are perhaps overstated.

(a) A drop of 13% in F1 for a gain of 2% in PPV is quite the trade-off. This is not as indicative of a strong predictor that could be used as the abstract claims. While the approach is novel and this is a good finding for a first attempt at such complex analysis, this is perhaps not as significant as the authors claim.

(b) In relation to this, there is a lack of situating these findings within the wider research landscape. For instance, the use of structure for predicting resistance has been done, for example, in PncA (https://academic.oup.com/jacamr/article/6/2/dlae037/7630603, https://www.sciencedirect.com/science/article/pii/S1476927125003664, https://www.nature.com/articles/s41598-020-58635-x) and in RpoB (https://www.nature.com/articles/s41598-020-74648-y). These, and other such works, should be acknowledged as the novelty of this work is perhaps not as stark as the authors present it to be.

We appreciate this comment and have made efforts to better situate our work in the wider context of structure-based prediction of antibiotic resistance. We have included description of and citation to these works and others in the introduction, results, and discussion. A differentiator between our work and previous is that we have trained a predictor across all proteins in the WHO catalogue of known resistance variants, not limited ourselves to a single protein at a time. We feel this makes the case that structure (and specifically proximity to known resistance-conferring variants) is a universally useful feature for resistance mutation prediction. We have also updated our abstract to reflect the exact performance of our method.

Introduction: “Protein three-dimensional structure has shown utility as an input feature for identifying resistance-conferring variants in known resistance-conferring proteins such as RpoB,[26,27] PncA [28–30], and AtpE.[31]While past work has sought to reannotate parts of the *M. tuberculosis* proteome with computationally predicted protein structures using older structure prediction methods,[32] we can now infer a protein structure for nearly every protein in the proteome using AlphaFold,[33] leading to new works examining the 3D location of mutations in known and suspected resistance-conferring proteins.[34,35]”

(3) The authors postulate that neutral AA substitutions would be randomly distributed in the protein structure and thus use random mutations as a negative control to simulate this neutral evolution. However, I am unsure if this is a true negative control for neutral evolution. The vast majority of residues would be under purifying selection, not neutral selection, especially in core proteins like rpoB and gyrA. Therefore, most of these residues would never be mutated in a real-world dataset. Therefore, you are not testing positive selection against neutral selection; you are testing positive against purifying, which will have a much stronger signal. This is likely to, in turn, overestimate the signal of positive selection. This would be better accounted for using a model of neutral evolution, although this is complex and perhaps outside the scope. Still, it needs to be made clear that these negative controls are not representative of neutral evolution.

The goal of our negative control was to simulate the random accumulation of amino acid substitutions without the effects of selection, which we had originally referred to as “neutral evolution” but is better described as “randomly accumulating substitutions.” We agree that in the absence of antibiotics, essential proteins like RpoB and GyrA are probably under purifying selection, and thus will have depletion of mutations in their hydrophobic cores. We have revised our wording in the results section “A protein-level statistic to test for mutational clustering” to make it clear that our negative control is that of randomly accumulating substitutions. We have included possible extension to more realistic evolutionary scenarios in the discussion.

As a side note, if we were to compare the observed mutation 3-D pattern to a purifying selection model, that may overestimate the signal compared with the randomly accumulating substitution model that we currently present in the manuscript. Purifying selection would tend to result in slower evolutionary rates than positive selection or randomly accumulation substitutions. So, a control based on purifying selection would have to have fewer mutations to account for the same evolutionary time, and this could lead to underestimating clustering.

(4) In a similar vein, the use of 15 Å as a cut-off for stating co-localisation feels quite arbitrary. The average radius of a globular protein is about 20 Å, so this could be quite a

large patch of a protein. I think it may be good to situate the cut-off for a 'single location' within a size estimator of the entire protein, as 15 Å could be a neighbourhood in a large protein, but be the whole protein for smaller ones.

We interrogated the use of 15 Å as a cutoff and found that it is indeed not very stringent, and functions more as a filter to remove the most egregious examples of proteins lacking single-location clustering. We include a new supplementary figure showing the number of significant hits as the cutoff is varied from 2 Å to 40 Å, and summarize these results in the main text. We note that we in fact find a weak negative relationship between protein length and the distance between the top two residues with highest G-score (R^2^ = 0.008, b = -3.1806, *p*-value = 0.048), the opposite of what would be expected under a scenario the distance between residues is simply driven by protein size and not a signal for clustering.

**Reviewer #2 (Public review):**

Summary:

This is an important study that, for the first time, systematically places the homoplastic genetic variation observed in the coding regions in a large collection of >31,000 *M. tuberculosis* samples into the protein structural context. This should be much more informative when, e.g. predicting antimicrobial resistance. The authors imaginatively apply the Getis-Ord score, which originated in geographical spatial analysis but has also been used in human disease to demonstrate that missense mutations in *M. tuberculosis* known to be associated with antimicrobial resistance are clustered in space. That they are able to consider almost all of the proteome using a large dataset of 31,000 *M. tuberculosis* complex clinical samples, which makes the evidence convincing.

Strengths:

To my knowledge, this is the first study to place the homoplastic missense mutations from a large clinical dataset into their protein structural context and attempt to look for clustering in space, which could be indicative of a recent evolutionary pressure, such as the use of antibiotics. The field usually only views resistance through the genetic paradigm, so it is delightful to see a structural paradigm being brought to bear, as this should, in theory, be much more informative, as protein structure is much closer to function. In addition, the dataset used is large (>31,000 clinical *M. tuberculosis* samples), and the authors are able to consider almost all of the ORFs (3,687/3,996) in the *M. tuberculosis* reference, and hence the analysis is comprehensive.

Weaknesses:

It is not apparent at the time of this review if the study could be reproduced by other researchers as e.g. whilst the authors state that the raw sequencing files (FASTQ) underpinning the dataset of 31,428 *M. tuberculosis* isolates can be downloaded the table in the Supplement containing the sample and accession identifiers contains rows that do not contain NCBI accessions e.g. '01R0685' or 'IDR 1600023875' or '1479144813357T181715lib5022nextseqn0035151bp' instead of the expected form e.g. 'SAMEA1016138'. I have searched the NCBI SRA using these terms and got no results, so they cannot be used to download any FASTQ files. There is also no information in the preprint on how the reads were processed (which is a complex process) and the dataset of SNPs subsequently built. One can trace back through the references, but I cannot find anywhere where one can download the SNP dataset, which would permit researchers to reproduce at least the latter stages of the work -- one obvious option would be to make the SNP dataset available. Likewise, the authors have constructed a "*M. tuberculosis* structureome", which would be very useful for the community but does not appear to be publicly available. At the time of the review, not all the GitHub repositories were public, so these points may have been rectified when that was corrected.

We have made a number of changes to improve reproducibility of the manuscript.

First, we have updated Table 1 with additional information to reflect the dataset of origin. While most of the isolates used are available from NCBI (94.5%), the remainder are from other sources. An additional 4.8% are exclusively from PATRIC (now the BV-BRC) and 0.4% are from ENA. Some of the isolates were originally named by their internal identifiers, not their NCBI BioSamples, which has been rectified. Of the three identifiers the reviewer cites, two were originally from Reseq-TB and are now listed with their NCBI BioSamples, and the third, 01R0685, corresponds to one isolate deposited with others in a single BioProject (https://www.ncbi.nlm.nih.gov/bioproject/?term=PRJEB26000; individual isolate available here: https://www.ncbi.nlm.nih.gov/biosample/10125872). We have clarified in the table metadata that the relevant search identifier.

Second, we have added a new section to our methods about the origins of the MTBC genomic data used in our manuscript, and describing the process of variant calling and SNP dataset construction.

Third, we have provided the data in a zenodo repository along with instructions for using the protein distance map files provided: 10.5281/zenodo.20766453

Lastly, we have ensured that the github is publicly available.

The authors correctly point out in the Introduction that supervised methods like GWAS or ML need datasets with matching genetic and phenotypic drug susceptibility data, which are much difficult/expensive to obtain, but don't then close the loop by comparing their results back to such supervised methods. They pick out RnJ as having previously been identified by a GWAS, but it would have provided a useful validation of their method to e.g. demonstrating that X% of the genes they identify were also identified by GWAS/ML studies, and therefore their method can achieve similar results but without having to collect pDST data.

We agree that this is a compelling possible extension of the work but due to time constraints have chosen not to pursue it for this manuscript.

Whilst the authors acknowledge that assuming all sites are equally likely to mutate in their random shuffling procedure is a shortcoming, a bigger weakness is, I suspect, that one should also only consider which amino acids could arise at each codon due to a SNP. Shuffling assumes any amino acid can arise at any codon which is only possible with multiple nucleotide changes, which is possible but highly unlikely.

Our approach is based on analyzing, in the wild-type protein structure, the 3D location at which mutations occur. In this calculation we do not consider the identity of the amino acid change per se. We have now clarified in the methods that we are not explicitly simulating biochemical change of the wild-type amino acid to any given mutant, rather we are analyzing the wild type amino acid in its structural context. We have added the following text in the methods section: In Computing inter-residue distances, “The EVcouplings Python package was used to compute the distance between wild-type amino acid residues in all protein structures”; in Computing the Getis-Ord score for clustering of homoplastic mutations, “The two values input to the Getis-Ord statistic computation are a per-residue score x, here the per-amino acid homoplasy score, and a weight matrix *W* that contains the inverse of the inter-residue distances computed from the wildtype amino acids”; and in Preparing GeO score calibration data, “Note that we do not recompute inter-residue distances when simulating mutations in an amino acid, as the distances used as input to GeO score are the wild-type inter-residue distances.” We hope this addresses the reviewer’s concern.

Finally, the authors implicitly assume that the mutations do not perturb the structure of the proteins, which is likely to be generally true for essential genes but less likely to be true for non-essential genes. This assumption underpins their entire approach and should be borne in mind when evaluating the results.

Our approach is based on analyzing, in the wild-type protein structure, the 3D location at which missense mutations occur and are observable in a naturally evolving population. It is true that we have not undertaken an analysis of whether any given mutation does or does not perturb the protein structure in which it occurs. However, location alone is a useful piece of information to analyze, as the location of naturally occurring mutations gives a readout of what types of mutations are allowed to persist under natural selection. Among our findings is that mutations display significant clustering even in non-essential genes, which we address in our discussion, “for proteins where mutations that lead to loss of function are known to cause resistance, such as PncA and RsmG (GidB), it is not necessarily expected to find clustering of mutations. We suspect that the observed clustering is due to mutations in a certain region of the protein being more likely to cause loss of function.”

**Recommendations for the authors:**

**Reviewer #1 (Recommendations for the authors):**

(1) It would be good if a more detailed description of the sequencing dataset origins were presented. The Supplementary Table 1, which is meant to hold these data, points to another paper, which in turn points to another paper which does not detail collection strategies for this data. This needs to be clear so that any bias in collection, which could over-inflate selection signals, can be assessed.

[From public reviews]

First, we have updated Table 1 with additional information to reflect the dataset of origin. While most of the isolates used are available from the NCBI (94.5%), the remainder are from other sources. An additional 4.8% are exclusively from PATRIC (now the BV-BRC) and 0.4% are from ENA. Some of the isolates were originally named by their internal identifiers, not their NCBI BioSamples, which has been rectified. Of the three identifiers the reviewer cites, two were originally from Reseq-TB and are now listed with their NCBI BioSamples, and the third, 01R0685, corresponds to one isolate deposited with others in a single BioProject (https://www.ncbi.nlm.nih.gov/bioproject/?term=PRJEB26000; individual isolate available here: https://www.ncbi.nlm.nih.gov/biosample/10125872). We have clarified in the table metadata that the relevant search identifier.

(2) Line 351: The exact download date is missing here.

We have added the exact download date

(3) Line 363: Did you mean lowest e-value? Higher would be worse.

Thank you for catching this, it is indeed the lowest e-value (confusion stemmed from looking at highest negative log e-value).

**Reviewer #2 (Recommendations for the authors):**

(1) The GitHub repo* was not public at the time of review (nor was it listed under user aggreen), so I could not check how reproducible the results are -- please make it public.

Absolutely, this has been addressed.

(2) The authors say "we envision structure being added as an additional feature in future work to predict resistance phenotypes from sequences" - this is not true, as some work has already been published predicting resistance in MBTC going back to 2019**

We have addressed this with wording changes and citations to the mentioned work (see public responses)

(3) Throughout the term 'non-synonymous' is used; that would include premature stop codons. Would 'missense' be more appropriate?

You’re correct in pointing out that the term “non-synonymous” is too general for what we mean in this paper. Our analysis included missense mutations and in-frame indels, but did not include premature stop (nonsense) mutations or frameshift mutations. So, the term missense (alone) is narrower than what we wish to convey. We have made clarifications throughout.

(4) The aminoglycosides are an important, albeit less used, class of antibiotics, and mutations arise in the ribosomal genes, e.g. rrs (which hence do not encode protein). They have therefore been excluded for obvious reasons, but it would help a reader from the tuberculosis field if this were acknowledged. Likewise, a reader might wonder why Rv0678 isn't in Figure 1 - I suspect it is because most of the samples were sequenced before the introduction of bedaquiline, but again, it would help if this were explained.

See response to (5)

(5) On a related note, it is not surprising that rpoC appears in Figure 1 due to its role in compensating for the fitness cost that arises when a rifamipicin-resistance mutation occurs in rpoB: obviously not central but a nice "oh yeah that makes sense" point for the reader if it were briefly mentioned.

These are both great points about the relevance of our results to the Mtb community. We have added an additional paragraph interpreting the results of Figure 1 that mentions the reason for the appearance of RpoC and Cas10, and non-appearance of non-coding genes and genes relevant to resistance to newly introduced and repurposed drugs.

(6) How is the "minimum coordinate difference" calculated? I assume all the structures are missing hydrogens as usual, so for two amino acids A and B, is it the smallest distance between any pair of heavy atoms from A and B? That would, I assume, introduce some bias for larger amino acids like Trp, or did you calculate from shared atoms like the backbone C_alpha atoms? That in turn will tend to make the distances a bit larger. It would be useful to know, as you explicitly mention a 1.5 nm threshold.

We have explained this in a new section of the results, “The EVcouplings Python package was used to compute the distance between amino acid residues in all protein structures [49]. The package calculates the distance between all heavy (non-hydrogen) atoms in residue *i* and residue *j*, then returns the minimum of those distances.”

(7) Given the reference used (H37Rv) is Lineage 4, one wonders about deeprooted/phylogenetic mutations, but then I suspect this sentence is doing a lot of that heavy-lifting: "We performed ancestral sequence reconstruction to determine the number of independent arisals of each mutation (homoplasy)". For the more general reader, it would be useful to touch on exactly what you mean and the importance of only considering homoplastic mutations.

We have expanded our explanation in this section to better make the case for the use of homoplastic variants in our analysis, “Because analyzing the frequency of alleles in a population can be biased by oversampling of particular lineages, and by evolutionary recency, we chose to analyze the number of independent arrivals of each mutation (homoplasy) rather than their population-level frequency. This ensures that more recent evolutionary events are not underrepresented due to lack of time to spread in the population. To accomplish this, we used a previously compiled a dataset of genomes of 31,428 isolates from the *Mycobacterium tuberculosis* complex (MTBC), with ancestral sequence reconstruction to determine the number of independent arrivals of each mutation (Supplementary Data 1).”

(8) Whilst this is true: "Evolution-based approaches are an alternative for finding variants associated with antibiotic resistance without requiring resistance phenotype data", the dataset used to, e.g. build the second edition of the WHO catalogue of resistanceassociated variants has >50,000 samples and therefore is larger than the dataset you have analysed here. The last time I looked, there were >100k *M. tuberculosis* samples in NCBI, and therefore, to be valid, your approach should really use more samples than are available with WGS and pDST data. I appreciate, however, that this will not be possible for this manuscript, but it is an obvious criticism.

We appreciate this critique and acknowledge that the number of isolates with both WGS and pDST has increased rapidly in recent years. The first edition of the WHO catalogue (2021) used 38,215 isolates, which increased to over 50k in the second edition. The dataset of homoplastic mutations on which we based this paper was originally published in 2021.

To address your comment, we have softened our assertions in the introduction about the utility of evolution-based approaches, and emphasize instead the different nature of the underlying signal, “Evolution-based approaches are an alternative for finding variants associated with antibiotic resistance by analyzing their mutational frequency and phylogenetic distribution”

(9) Minor point, but the second sentence in the Introduction ignores that one can diagnose MDR-TB using phenotypic methods as well as genetic methods.

We have added an additional citation and mention of laboratory phenotypic methods.

(10) There are a few typos: "genic" "G-sore"

Addressed.
